# Menopause is a physiological progression event for women with multiple sclerosis: No

**DOI:** 10.1177/13524585261429806

**Published:** 2026-04-01

**Authors:** Vilija G Jokubaitis

**Affiliations:** Department of Neuroscience, School of Translational Medicine, Monash University, Melbourne, VIC, Australia; Department of Neurology, Alfred Health, Melbourne, VIC, Australia

Midlife brings the convergence of two major transitions for women: biological ageing and reproductive ageing. Biological ageing is characterised by a shift towards inflammaging and cellular senescence, with broad metabolic, paracrine and endocrine effects, manifested as physiological decline across a number of health conditions including in multiple sclerosis (MS).^1^ Reproductive ageing sees the transition to the complete cessation of ovarian function, and relative sex hormone insufficiency, accompanied by increased risk of comorbid conditions including cardiovascular disease (CVD), osteoporosis and an increased risk of certain cancers.

Sex hormones, particularly oestrogen, have long been considered important modulators of MS biology. The shift in sex ratio from 1:1 female to male prior to puberty, to approximately 3:1 after puberty, the marked reduction in relapse activity during pregnancy, and experimental evidence demonstrating neuroprotective and immunomodulatory actions of oestrogen, progesterone and androgens together support a role for sex hormones in shaping MS outcomes.^2^ This has led to the intuitively appealing hypothesis that the decline in sex hormones at menopause may accelerate neurodegeneration and disability progression.

A growing body of empirical evidence, however, challenges this hypothesis. Several studies have sought to determine whether sex hormone insufficiency at midlife contributes to disability worsening in women with MS.^3–7^ Recent work by Silverman reported an age-adjusted increase in neurofilament and a decline in the MS functional composite score (MSFC, driven by Expanded Disability Status Scale (EDSS) change), at menopause suggesting an association with disability progression.^5^ Notably, however, no inflection point in the MSFC was identified, and the observed effects were modest, realised over a 15-year time frame.

In contrast, in the largest prospective study to date, including 987 women from the MSBase Registry, menopause was not found to increase the risk of confirmed disability progression nor transition to secondary progressive MS.^3^ Within-person modelling of EDSS trajectories similarly failed to reveal a discrete menopausal inflection point.^3^ Independent cohorts reinforce these findings. In the Barcelona CIS cohort, menopause did not modify disability trajectories after adjusting for chronological age and disease duration.^4^ A Norwegian cohort of 559 peri- and postmenopausal women similarly showed no menopausal effect on disability after robust adjustment for age.^6^

Genetic causal inference aligns with these null findings. Using data from multiple large consortia (ReproGen, UK Biobank, IMSGC), Sun and colleagues found no causal effect of age at menopause on MS severity.^8^ Across these large, well-powered studies, biological ageing, not reproductive ageing, consistently emerges as the dominant driver of disability accumulation at midlife.

The idea that menopause directly accelerates progression rests on the known neuroprotective and immunomodulatory effects of oestrogen and progesterone.^2^ Although these hormones unquestionably influence neuroinflammatory and reparative processes, their effects are likely context-dependent and embedded within broader ageing biology.

An alternative and more compelling explanation for the apparent midlife acceleration in disability is the metabolic and body composition changes that accompany ageing. Weight gain is common at midlife in both sexes, with typical increases ranging between 0.3 and 0.5 kg per year between the ages of 40 and 60, after which weight may stabilise, or even decline fractionally after the age of 60.^9^ Women also experience accelerated visceral adiposity in the late perimenopausal period (0–2 years prior to the last menstrual period).^10^ Hormonal fluctuations occur throughout the perimenopausal transition. Importantly, more than 44% of women experience increases in estradiol during the late perimenopausal period,^11^ underscoring that the menopausal transition is not an oestrogen-deplete state.

In the MSBase analysis, additional modelling examining early and late perimenopause also showed no association with disability outcomes after adjusting for age, disease duration and EDSS score.^3^ These observations challenge the centrality of the MS-oestrogen hypothesis in midlife. The temporal overlap between midlife weight gain and disability progression instead suggests that metabolic ageing may mediate much of the perceived menopause-related worsening. Biological ageing in the fifth and sixth decades represents an inflection point in disability progression for all genders.^1^

Increased visceral adiposity is biologically relevant: it drives systemic inflammation, induces mitochondrial stress, alters endocrine signalling and is associated with higher serum neurofilament light (sNfL) concentrations.¹ Higher body mass index (BMI) is also an established predictor of faster disability accumulation. Thus, metabolic change, not reproductive ageing, may be the modifiable factor most meaningfully linked to midlife progression.

Midlife represents a period of physiological and psychosocial transition for women with MS. Menopause may amplify symptoms, affect mood and alter quality of life, but current evidence does not support the view that it fundamentally alters MS disease biology, or represents a physiological ‘cliff’. Rather, disability progression at midlife is more parsimoniously explained by biological ageing and the associated metabolic, paracrine and systemic effects that drive comorbidity burden.

The effects of biological and reproductive ageing undoubtedly intersect, and disentangling these influences remains a critical research need. Large, prospective, longitudinal studies incorporating blood, magnetic resonance imaging (MRI), cognitive and digital biomarkers across both women and men will be essential to accurately characterise how these processes interact to drive disability progression in MS.

Clinically, reframing menopause as a *life stage*, not a *disease stage*, refocuses attention on modifiable determinants of progression. This includes optimising metabolic health, promoting physical activity, managing comorbidities, addressing sleep and mood disorders, and ensuring sustained access to high-efficacy disease-modifying therapies. Concurrently, menopausal symptoms should be treated to preserve quality of life and functional independence.

Rather than attributing midlife worsening to reproductive ageing, we must address the twin engines of progression: ageing biology and midlife metabolic risk. Recognising this distinction opens opportunities for targeted intervention and more meaningful improvements in long-term outcomes for women with MS.
